# Revisiting Rab7 Functions in Mammalian Autophagy: Rab7 Knockout Studies

**DOI:** 10.3390/cells7110215

**Published:** 2018-11-19

**Authors:** Yoshihiko Kuchitsu, Mitsunori Fukuda

**Affiliations:** Laboratory of Membrane Trafficking Mechanisms, Department of Integrative Life Sciences, Graduate School of Life Sciences, Tohoku University, Aobayama, Aoba-ku, Sendai, Miyagi 980-8578, Japan; y.kuchi1212@gmail.com

**Keywords:** autophagy, autolysosome maturation, glutamine starvation, lysosome, Rab7/Ypt7

## Abstract

Rab7 (or Ypt7 in yeast) is one of the well-characterized members of the Rab family small GTPases, which serve as master regulators of membrane trafficking in eukaryotes. It localizes to late endosomes and lysosomes and has multiple functions in the autophagic pathway as well as in the endocytic pathway. Because Rab7/Ypt7 has previously been shown to regulate the autophagosome-lysosome fusion step in yeast and fruit flies (i.e., autophagosome accumulation has been observed in both Ypt7-knockout [KO] yeast and Rab7-knockdown fruit flies), it is widely assumed that Rab7 regulates the autophagosome-lysosome fusion step in mammals. A recent analysis of Rab7-KO mammalian cultured cells, however, has revealed that Rab7 is essential for autolysosome maturation (i.e., autolysosome accumulation has been observed in Rab7-KO cells), but not for autophagosome-lysosome fusion, under nutrient-rich conditions. Thus, although Rab7/Ypt7 itself is essential for the proper progression of autophagy in eukaryotes, the function of Rab7/Ypt7 in autophagy in yeast/fruit flies and mammals must be different. In this review article, we describe novel roles of Rab7 in mammalian autophagy and discuss its functional diversification during evolution.

## 1. Introduction

Rab small GTPases constitute the largest subfamily of the Ras superfamily, and they are well conserved in all eukaryotes, from yeast to mammals. They play critical roles in various membrane trafficking steps, including vesicle formation, transport, docking, and fusion, and they control the proper progression of various membrane trafficking events, including endocytosis, exocytosis, and autophagy [1,2,3,4]. The same as other Ras-like small GTPases, Rabs function as switch molecules by cycling between two nucleotide-bound states, a GDP-bound inactive state and a GTP-bound active state. Active Rabs are recruited to specific membrane compartments and regulate their trafficking through interaction with specific effector molecules [5]. Approximately 60 different Rab isoforms are present in mammals, and most of them have different subcellular localizations.

**Figure 1:**
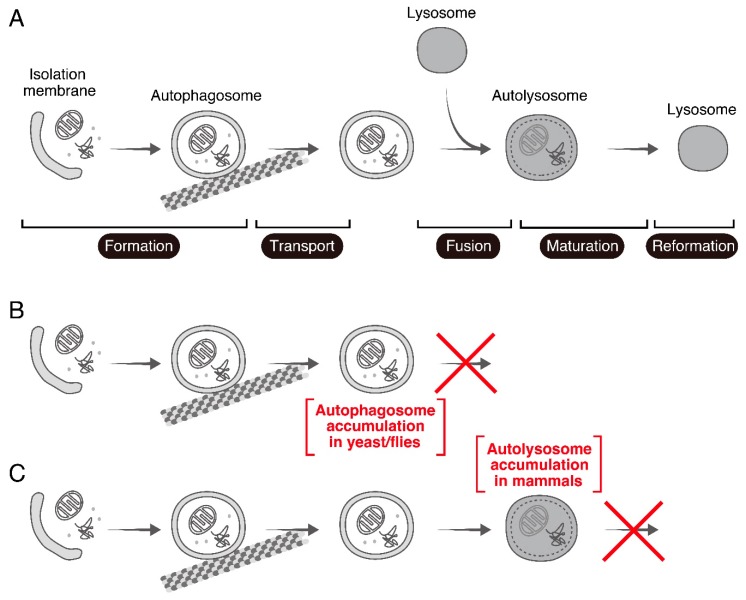
Overview of autophagy and the autophagic phenotypes reported in Rab7-KO yeast/fruit flies and mammals. (**A**) Overview of autophagy. Upon induction of autophagy by stresses such as nutrient starvation, an isolation membrane emerges in the cytoplasm and sequesters cytoplasmic contents to form an autophagosome (formation step). The autophagosome is delivered to the perinuclear region along microtubules (transport step), where it fuses with a lysosome to form an autolysosome (fusion step). Energy is supplied by degradation of the cargos sequestered in the autolysosome (maturation step), and, finally, a new lysosome is formed (reformation step). (**B**) Accumulation of autophagosomes in Ypt7-KO yeast and Rab7-KD fruit flies, suggesting that Rab7/Ypt7 regulates the autophagosome-lysosome fusion step. (**C**) Accumulation of autolysosomes in Rab7-KO mammalian cultured cells, suggesting that mammalian Rab7 regulates the autolysosome maturation step.

Rab7 (or Ypt7 in yeast) is one of the “housekeeping” Rabs that are conserved in all eukaryotes [6], and it has been shown to play pivotal roles in late endosome trafficking and lysosome biogenesis [7,8]. In addition to being involved in the endocytic pathway, Rab7/Ypt7 is known to be involved in the autophagic pathway [9], an intracellular degradation system that is also conserved from yeast to mammals. Macroautophagy (unless otherwise specified, simply referred to as autophagy below) plays important physiological roles in response to various stresses such as nutrient starvation [10]. During autophagy isolation membranes (also called phagophores) emerge in the cytoplasm, elongate, and sequester cytosolic contents to form double-membrane-bound spherical structures called autophagosomes. The autophagosomes are transported to the perinuclear region in a microtubule-dependent manner and fuse with lysosomes to form hybrid organelles called autolysosomes. After degradation of the contents, lysosomes are reformed from the autolysosomes [11] (Figure 1A). Rab7 has been shown to regulate the autophagosome-lysosome fusion step in the autophagy process in yeast and fruit flies (Figure 1B) [12,13,14]. Since several studies conducted by using small interfering RNA (siRNA)-mediated knockdown (KD) of Rab7 and/or overexpression of a dominant negative mutant (i.e., GDP-fixed form) of Rab7 in mammalian cells have supported this finding [15,16], it is widely assumed that mammalian Rab7 also regulates the autophagosome-lysosome fusion step. However, a recent analysis of Rab7 knockout (KO) mammalian cultured cells generated by using the CRISPR/Cas9 system unexpectedly showed that loss of Rab7 phenotypes in mammals is clearly different from the loss of phenotype observed in yeast and fruit flies, and that mammalian Rab7 is involved in autolysosome maturation under nutrient-rich conditions [17] (Figure 1C). In this review article, we provide an up to date overview of studies on the roles of Rab7 in mammalian autophagy, especially highlighting the difference between its roles in autophagy in yeast/fruit flies and in mammals.

## 2. Proposed Roles of Rab7 in Autophagy: Previous Knockdown and Overexpression Studies

### 2.1. Roles of Rab7 in Bidirectional Transport of Autophagosomes on Microtubules

**Figure 2:**
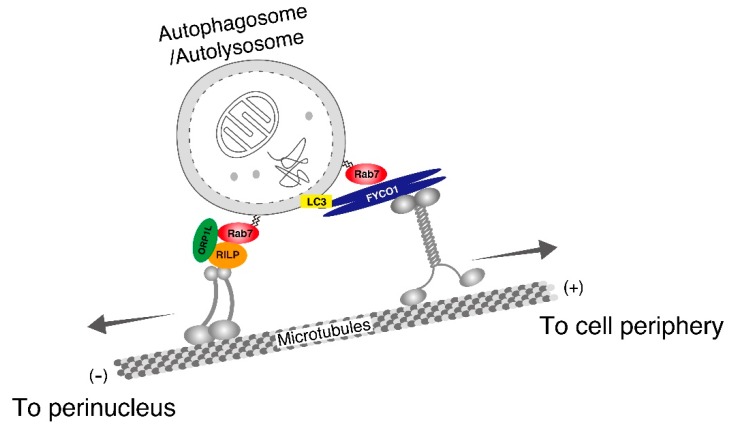
Proposed model of the roles of Rab7 in bidirectional transport of autophagosomes on microtubules. Rab7 links autophagosomes to kinesin through FYCO1 and facilitates microtubule-dependent plus-end-directed transport towards the cell periphery [19]. Rab7 also mediates microtubule-dependent minus-end-directed transport towards the perinuclear region through interaction with its effectors, ORP1L and RILP, which binds to the dynein-dynactin complex [20,21].

Although autophagosomes appear to be formed randomly throughout the cytoplasm, lysosomes are mainly localized in the perinuclear region. If autophagosomes are, in fact, formed randomly throughout the cytoplasm, then they must be delivered to the perinuclear region along microtubules in order to be able to eliminate their cargos [18]. Microtubule-dependent transport occurs bidirectionally through specific motor proteins: The minus-end-directed dynein-dynactin motor complex transports its cargos to the perinuclear region, whereas plus-end-directed kinesin motor proteins transport their cargos towards the cell periphery. Rab7 has previously been shown to regulate the proper bidirectional transport of autophagosomes (or autolysosomes) through interaction with its specific effectors [19,20,21]. The plus-end-directed transport of autophagosomes is regulated by Rab7 and its effector FYCO1 (FYVE and coiled-coil domain-containing 1) [19]. In addition to Rab7 binding, FYCO1 is likely to interact with kinesin in its N-terminal region, and it also interacts with LC3, which is specifically localized to autophagosomes, and PI3P (phosphatidylinositol 3-phosphate) in its C-terminal region [19]. Thus, FYCO1 functions as a linker between a kinesin motor and LC3-resident autophagosomes during plus-end-directed autophagosome transport (Figure 2). Rab7 also regulates minus-end-directed autophagosome transport through the formation of a complex with RILP (Rab-interacting lysosomal protein), a cholesterol sensor ORP1L (oxysterol-binding protein-related protein 1; also known as OSBPL1A), and dynein [20,21] (Figure 2).

### 2.2. Role of Rab7 in Autophagosome-Lysosome Fusion

After autophagosomes are formed and moved to the perinuclear region, they fuse with lysosomes (the resulting hybrid organelles are called autolysosomes), which enables degradation of their content. Since late endosomes fuse with lysosomes and thereby enable degradation in the endocytic pathway, it seems reasonable to assume that the machinery used in the autophagosome-lysosome fusion step is the same or similar to the machinery used in the late endosome-lysosome fusion step. Actually, Rab7, which is localized to late endosomes and lysosomes and controls the late endosome-lysosome fusion step [7,8], has been shown to play a pivotal role in the autophagosome-lysosome fusion step [12,13,14]. Consistent with the above assumption, Rab7 deficiency in yeast and fruit flies has been shown to result in massive accumulation of autophagosomes [12,13,14].

Involvement of mammalian Rab7 in autophagy has been investigated by siRNA-mediated KD of Rab7 and/or overexpression of its dominant negative mutant [15,16]. Rab7 appeared to be recruited to autophagosomes rather than to late endosomes in these studies, because Rab7 recruitment to autophagosomes was detected before fusion with LBPA (lysobisphosphatidic acid; late endosome marker)-positive or Lamp1 (lysosome marker)-positive compartments [15,16]. Furthermore, because there were few LC3- and Lamp1-double-positive autolysosomes in the perinuclear region, functional ablation of Rab7 was conducted to inhibit the autolysosome formation instead of affecting autophagosome formation. Thus, until recently Rab7 had widely been thought to regulate the autophagosome-lysosome fusion step in mammals, the same as it does in yeast and fruit flies.

## 3. Rab7 Knockout Studies in Mammalian Cultured Cells and Mice

### 3.1. Analysis of Rab7 Knockout in Mammalian Cultured Cells: A Novel Role of Rab7 in Autolysosome Maturation and the Discovery of Glutamine-Starvation-Induced Autolysosome Clearance

**Figure 3:**
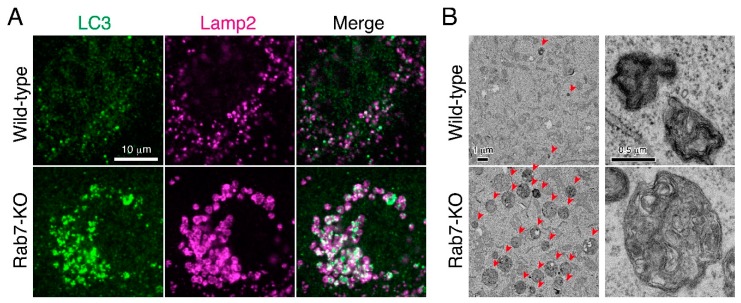
Abnormal autolysosome accumulation in Rab7-KO MDCK-II cells. (**A**) Accumulation of enlarged LC3 (autophagosome marker)- and Lamp2 (lysosome marker)-double positive autolysosomes in Rab7-KO cells [17]. By contrast, only a few LC3-positive puncta were observed in wild-type cells, and a larger portion of the LC3 puncta is negative for Lamp2 (i.e., autophagosomes). (**B**) Ultrastructure of the accumulated autolysosomes in Rab7-KO cells. Transmission electron microscopic images of autolysosomes (or lysosomes) with high-electron-dense single-membrane structures in Rab7-KO cells. The arrowheads point to autolysosomes (or lysosomes), which were dramatically increased in Rab7-KO cells. The images on the right show typical autolysosome (or lysosome) structures in the wild-type cells and Rab7-KO cells.

**Figure 4:**
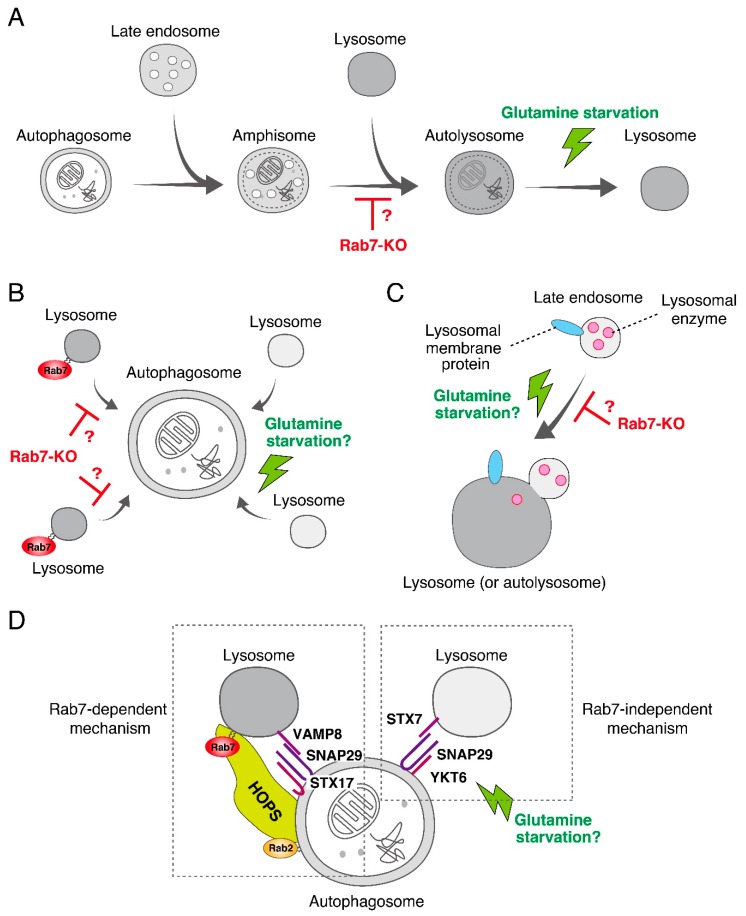
Possible models of Rab7-dependent and -independent autolysosome maturation in mammalian cells. (**A**) Rab7 regulates the amphisome-lysosome fusion step. It has been suggested that during the process of autophagy, the autophagosome sequentially fuses with an endosome and with a lysosome to form an amphisome and an autolysosome, respectively. Loss of Rab7 would cause autolysosome accumulation, and glutamine starvation would rapidly induce lysosome fusion to the amphisome. (**B**) Rab7 regulates the efficiency of autophagosome-lysosome fusion. In this model, an autophagosome in a mammalian cell fuses with multiple lysosomes thereby enabling complete degradation of its content. Loss of Rab7 should result in a decrease in the number of lysosomes fused to the autophagosome and lead to autolysosome accumulation. Glutamine starvation may rapidly induce fusion of additional lysosomes to the autophagosome and result in complete degradation. (**C**) Rab7 regulates lysosomal functions by transporting lysosomal enzymes and/or lysosomal membrane proteins from the late endosome to the lysosome. Because delivery of lysosomal enzymes to lysosomes is partially impaired in Rab7-KO cells [17,25,26], this process may be upregulated by glutamine starvation. (**D**) Two independent SNARE complexes, the syntaxin17 (STX17)-SNAP29-VAMP8 complex and the syntaxin7 (STX7)-SNAP29-YKT6 complex, control autophagosome-lysosome fusion [29], consistent with the model shown in (B). Rab7 cooperates with the STX17-SNAP29-VAMP8 complex together with Rab2 and the HOPS complex in mediating autophagosome-lysosome fusion [13]. By contrast, the STX7-SNAP29-YKT6 complex is likely to be Rab7-independent and partially mediates the fusion even in the absence of Rab7. Future investigation will be necessary to determine whether glutamine starvation activates the function of the STX7-SNAP29-YKT6 complex. In contrast to this model, however, it has recently been proposed that two SNARE complexes, STX17-SNAP29-YKT6 and STX17-SNAP29-VAMP7/8, in fruit flies sequentially regulate autophagosome-lysosome fusion by replacing YKT6 with VAMP7/8 [30].

To confirm the widely assumed additional role of mammalian Rab7 as a critical regulator in autophagosome-lysosome fusion as described above [15,16], we recently knocked out Rab7 in MDCK-II (Madin-Darby canine kidney II) cells by using the CRISPR/Cas9 system. Although the number of LC3 dots was dramatically increased in Rab7-KO cells, they were unexpectedly found to be positive for Lamp2 (a lysosome marker), indicating that autolysosomes rather than autophagosomes (i.e., LC3-positive and Lamp2-negative structures) accumulated under nutrient-rich conditions (Figure 3A) [17]. Consistent with this finding, a transmission electron microscopic analysis revealed the presence of a number of electron-dense autolysosomes, which often contained numerous membranous structures, in Rab7-KO cells in comparison with wild-type cells (Figure 3B). By contrast, virtually no accumulation of double membrane structures (i.e., autophagosomes) was observed in the Rab7-KO cells under nutrient-rich conditions. Thus, autophagosome-lysosome fusion must occur even in the absence of Rab7, and mammalian Rab7 must instead be required for the subsequent autolysosome maturation step [17]. Autolysosome accumulation is not a specific event in Rab7-KO MDCK-II cells, because the same phenomenon was also observed in Rab7-KD HeLa cells. How Rab7 regulates autolysosome maturation is unknown, but one or more of the following three mechanisms would explain the function of Rab7 in the maturation step (Figure 4). (1) Rab7 regulates the amphisome-lysosome fusion step [16]. Autophagosomes are also known to fuse with endosomes to form hybrid organelles called amphisomes before fusing with lysosomes [22,23], and thus loss of Rab7 would result in accumulation of partially degraded amphisome-like structures (Figure 3B and Figure 4A). (2) Rab7 regulates the efficiency of autophagosome-lysosome fusion (Figure 4B). Live-cell imaging has recently shown that the contents of a single autophagosome are degraded by fusing with multiple lysosomes [24]. Loss of Rab7 would therefore result in a decrease in the number of lysosomes that fuse with autophagosomes, and consequently the autolysosome maturation process would be delayed (Figure 4B). (3) Rab7 regulates the transport of lysosomal enzymes and/or lysosomal membrane proteins (e.g., vacuolar ATPase V0 subunit) to lysosomes (Figure 4C). Actually, the amount of the mature form of cathepsin B has been found to be reduced in Rab7-KO cells and its immature form to be abnormally secreted into the culture medium [17,25,26]. Thus, lysosomal enzyme activity in Rab7-KO cells may be impaired, which would lead to autolysosome accumulation, however, cathepsin B activity, monitored with Magic Red, and lysosomal pH, monitored with LysoTracker Red, in Rab7-KO cells appeared to be normal [17].

Another unexpected finding obtained in analyses of Rab7-KO cells was that Rab7 is dispensable for starvation-induced autophagy. The autolysosome accumulation phenotype of Rab7-KO cells described above was observed only under nutrient-rich conditions, and the autolysosomes that had accumulated in Rab7-KO cells were rapidly cleared by a brief period of nutrient starvation [17]. Actually, normal flux of LC3, an indicator of autophagic activity, was observed under starved conditions even in the absence of Rab7. Intriguingly, the starvation-induced autolysosome clearance in Rab7-KO cells is dependent on the presence of an amino acid, specifically glutamine, and not on the presence of serum or glucose [17]. The same phenomenon was also observed in Rab7-KD HeLa cells, suggesting that mammalian Rab7 regulates autolysosome maturation only under nutrient-rich conditions and that an unidentified Rab7-independent mechanism(s) must be activated for autolysosome clearance to occur under nutrient (glutamine)-starved conditions. This mechanism is clearly different from the known TFEB-mediated lysosome activation [27,28], because neither actinomycin D (a transcription inhibitor) nor cycloheximide (a translation inhibitor) inhibited glutamine-starvation-induced autolysosome clearance in Rab7-KO cells. Furthermore, mTORC1 inactivation is not involved in this process, because Torin 2, a specific inhibitor of mTORC1, had no effect on autolysosome clearance in Rab7-KO cells [17].

More importantly, glutamine-starvation-induced autolysosome clearance was retained in wild-type MDCK-II cells as well as in HeLa cells, indicating that the Rab7-independent mechanism responsible for starvation-induced autolysosome clearance is not a backup mechanism that is activated only in the absence of Rab7, and that it must function normally in wild-type cells. How glutamine starvation facilitates autolysosome clearance is unknown, but lysosomal enzyme activities are expected to be activated by an as yet unidentified mechanism(s). Glutamine starvation may promote amphisome-lysosome fusion, additional fusion of lysosomes with autophagosomes, and/or transport of lysosomal enzymes/proteins to lysosomes in a Rab7-independent manner (Figure 4).

### 3.2. Analysis of Rab7 Knockout Mice

Since Rab7-KO mice die at a very early embryonic stage [31,32], whereas mice lacking essential autophagy proteins, including Atg3, Atg5, and Atg16L1, are born normally but die in the neonatal period due to defects in macroautophagy during neonatal starvation [33], deficient macroautophagy is unlikely to be a primary cause of the embryonic lethality of Rab7-KO mice. Actually, Rab7 has been reported to be essential for “microautophagy”, another type of autophagy that is mediated by direct engulfment of cytosolic cargos with lysosomes [31]. During early embryogenesis, nutrients and signaling molecules enter the embryo through an epithelial tissue called visceral endoderm (VE). The material that has entered VE cells is delivered to lysosomes, and unusually large apical vacuoles are formed as a result. Because Rab7 is required for the assembly of large apical vacuoles, a massive number of small vesicles, which morphologically resembled endosomes, together with a few large apical vacuoles were observed in Rab7-deficient VE cells [31]. However, no autophagosome or autolysosome accumulation was reported in the VE cells of Rab7-KO mice.

Several immune cell-conditional and pancreatic cell-conditional Rab7-KO mice have also been reported [32,34,35]. Autophagic flux monitored with LC3-II has been found to be blocked in Rab7-deficient T cells and MEFs (embryonic fibroblasts) under nutrient-rich conditions [32], consistent with the findings in Rab7-KO MDCK-II cells and Rab7-KD HeLa cells [17]. Moreover, a block of autophagy flux has also been observed in Rab7-KO pancreatic cells, although a transmission electron microscopic analysis revealed accumulation of autophagosomes rather than autolysosomes [35]. By contrast, normal induction of autophagy and normal LC3-II flux were observed in Rab7-deficient B cells [34], suggesting the presence of a compensatory mechanism. Such discrepancies in the Rab7-KO phenotypes may be explained by different contributions of Rab7 to macroautophagy in different cell types (see Perspectives below).

## 4. Concluding Remarks and Perspectives

In this review article, we have summarized recent findings regarding the functions of Rab7 in mammalian autophagy, especially focusing on Rab7 KO in cultured cells and in mice. Although mammalian Rab7 was originally proposed to regulate autophagy, specifically at the autophagosome transport step on microtubules and the autophagosome-lysosome fusion step [12,13,14,15,16,18,19,20,21], our recent KO analyses revealed that Rab7 is involved in the autolysosome maturation step in several types of mammalian cultured cells [17], indicating that Rab7 is not essential for the autophagosome–lysosome fusion step in mammals. Moreover, no abnormal autophagosome or autolysosome distribution in Rab7-KO cells has been reported thus far [17,32,34,35]. A more precise analysis such as by live-cell autophagosome imaging will be necessary to determine whether autophagosome/autolysosome transport is actually impaired in Rab7-KO cells.

Why are the roles of Rab7 in autophagy in yeast/fruit flies and mammals different? The simplest answer is the existence of the Rab7-independent autophagosome-lysosome fusion mechanism in mammals. The most likely candidate for this mechanism is a compensatory mechanism involving other Rabs, because mammals contain ~60 different Rab isoforms, many more than ~10 isoforms in yeast and ~30 isoforms in fruit flies [6]. In fact, at least two Rab7-related isoforms, Rab7B (also called Rab42) and Rab7L (also called Rab29), are present in mammals. Generation and analysis of Rab7/7B/7L triple-KO cells in the future will be necessary to identify functional redundancies among the Rab7-related isoforms during autophagy. Alternatively, an as yet additional unidentified Rab-independent mechanism that mediates autophagosome-lysosome fusion may exist. We have especially noted a recent report stating that two SNARE complexes, i.e., a syntaxin17-SNAP29-VAMP8 complex and a syntaxin7-SNAP29-YKT6 complex, independently regulate autophagosome-lysosome fusion [29], and the former complex has been shown to mediate autophagosome-lysosome fusion together with Rab7 and a HOPS tethering complex in fruit flies [13] (Figure 4D). Thus, it is highly possible that the latter complex, which presumably does not depend on any Rabs, mediates autophagosome-lysosome fusion even in the absence of Rab7. It will be interesting to determine whether autophagosome-lysosome fusion mediated by the latter complex is activated by glutamine starvation in the future, because normal autophagy flux is observed under starved conditions. It is likely that the contributions of two independent fusion mechanisms to autophagy differ, i.e., that they are fully or partly redundant, or that one mechanism is missing in some organisms, tissues, or cell-types, and that could explain why the Rab7-KO phenotypes differ among organisms [12,13,14,17] or different cell-types in Rab7-KO mice [34,35].

In addition to being involved in macroautophagy, Rab7 has been shown to be involved in several forms of selective autophagy in mammals [36,37,38,39]. For example, Rab7 is required for the formation of GAS (group A *Streptococcus*)-containing autophagosome-like structures (a process called xenophagy) that sequester invading pathogenic bacteria [36]. Rab7 also functions in the elongation of isolation membranes during mitophagy [37,38,39]. In both cases, Rab7 plays a crucial role in the early stage of autophagy (i.e., formation step) rather than in the late stage (i.e., maturation step). Rab7 may mediate fusion between elongating autophagosomes and endocytic compartments that leads to sequestration of large bacteria and mitochondria before ultimate fusion with lysosomes. However, since Rab7 involvement in xenophagy and mitophagy has mainly been demonstrated by siRNA-mediated KD of Rab7, it will be necessary to determine whether Rab7 KO also causes defects in these forms of selective autophagy.

Because mutations in the *RAB7* gene cause Charcot-Marie-Tooth type 2B (CMT2B) peripheral neuropathy [40], it will also be important to investigate whether CMT2B-associated Rab7 mutations are directly involved in autolysosome accumulation. Actually, overexpression of CMT2B-associated Rab7 mutants in mammalian cultured cells has been shown to impair autophagic flux [41]. However, the exact mechanism underlying the disease phenotype has not been fully understood, and further investigation is needed to clarify the impact of CMT2B-causing mutations on autolysosome maturation. Future re-investigation of the proposed functions of Rab7 in the endocytic pathway (e.g., in EGFR [epidermal growth factor receptor] degradation) [7,8] and in selective autophagy by using Rab7-KO cells will reveal the real functions of Rab7 in mammals, which would lead to a better understanding of the impaired Rab7 function(s) that directly causes CMT2B.

## References

[1] Fukuda M. (2008). Regulation of secretory vesicle traffic by Rab small GTPases. Cell. Mol. Life Sci..

[2] Hutagalung A.H., Novick P.J. (2011). Role of Rab GTPases in membrane traffic and cell physiology. Physiol. Rev..

[3] Zhen Y., Stenmark H. (2015). Cellular functions of Rab GTPases at a glance. J. Cell Sci..

[4] Pfeffer S.R. (2017). Rab GTPases: Master regulators that establish the secretory and endocytic pathways. Mol. Biol. Cell.

[5] Pfeffer S.R. (2013). Rab GTPase regulation of membrane identity. Curr. Opin. Cell Biol..

[6] Diekmann Y., Seixas E., Gouw M., Tavares-Cadete F., Seabra M.C., Pereira-Leal J.B. (2011). Thousands of Rab GTPases for the cell biologist. PLoS Comput. Biol..

[7] Wang T., Ming Z., Xiaochun W., Hong W. (2011). Rab7: Role of its protein interaction cascades in endo-lysosomal traffic. Cell. Signal..

[8] Guerra F., Bucci C. (2016). Multiple roles of the small GTPase Rab7. Cells.

[9] Hyttinen J.M.T., Niittykoski M., Salminen A., Kaarniranta K. (2013). Maturation of autophagosomes and endosomes: A key role for Rab7. Biochim. Biophys. Acta.

[10] Nakatogawa H., Suzuki K., Kamada Y., Ohsumi Y. (2009). Dynamics and diversity in autophagy mechanisms: Lessons from yeast. Nat. Rev. Mol. Cell Biol..

[11] Yu L., McPhee C.K., Zheng L., Mardones G.A., Rong Y., Peng J., Mi N., Zhao Y., Liu Z., Wan F. (2010). Termination of autophagy and reformation of lysosomes regulated by mTOR. Nature.

[12] Kirisako T., Baba M., Ishihara N., Miyazawa K., Ohsumi M., Yoshimori T., Noda T., Ohsumi Y. (1999). Formation process of autophagosome is traced with Apg8/Aut7p in yeast. J. Cell Biol..

[13] Fujita N., Huang W., Lin T.H., Groulx J.F., Jean S., Nguyen J., Kuchitsu Y., Koyama-Honda I., Mizushima N., Fukuda M. (2017). Genetic screen in Drosophila muscle identifies autophagy-mediated T-tubule remodeling and a Rab2 role in autophagy. eLife.

[14] Péter L., Tóth S., Benkö P., Lakatos Z., Boda A., Glatz G., Zobel M., Bisi S., Heged K., Takáts S. (2017). Rab2 promotes autophagic and endocytic lysosomal degradation. J. Cell Biol..

[15] Gutierrez M.G., Munafó D.B., Berón W., Colombo M.I. (2004). Rab7 is required for the normal progression of the autophagic pathway in mammalian cells. J. Cell Sci..

[16] Jäger S., Bucci C., Tanida I., Ueno T., Kominami E., Saftig P., Eskelinen E.-L. (2004). Role for Rab7 in maturation of late autophagic vacuoles. J. Cell Sci..

[17] Kuchitsu Y., Homma Y., Fujita N., Fukuda M. (2018). Rab7 knockout unveils regulated autolysosome maturation induced by glutamine starvation. J. Cell Sci..

[18] Kimura S., Noda T., Yoshimori T. (2008). Dynein-dependent movement of autophagosomes mediates efficient encounters with lysosomes. Cell Struct. Funct..

[19] Pankiv S., Alemu E.A., Brech A., Bruun J.A., Lamark T., Overvatn A., Bjorkoy G., Johansen T. (2010). FYCO1 is a Rab7 effector that binds to LC3 and PI3P to mediate microtubule plus end-directed vesicle transport. J. Cell Biol..

[20] Jordens I., Fernandez-Borja M., Marsman M., Dusseljee S., Janssen L., Calafat J., Janssen H., Wubbolts R., Neefjes J. (2001). The Rab7 effector protein RILP controls lysosomal transport by inducing the recruitment of dynein-dynactin motors. Curr. Biol..

[21] Wijdeven R.H., Janssen H., Nahidiazar L., Janssen L., Jalink K., Berlin I., Neefjes J. (2016). Cholesterol and ORP1L-mediated ER contact sites control autophagosome transport and fusion with the endocytic pathway. Nat. Commun..

[22] Shen H.-M., Mizushima N. (2014). At the end of the autophagic road: An emerging understanding of lysosomal functions in autophagy. Trends Biochem. Sci..

[23] Nakamura S., Yoshimori T. (2017). New insights into autophagosome–lysosome fusion. J. Cell Sci..

[24] Tsuboyama K., Koyama-Honda I., Sakamaki Y., Koike M., Morishita H., Mizushima N. (2016). The ATG conjugation systems are important for degradation of the inner autophagosomal membrane. Science.

[25] Modica G., Skorobogata O., Sauvageau E., Vissa A., Yip C.M., Kim P.K., Wurtele H., Lefrancois S. (2017). Rab7 palmitoylation is required for efficient endosome-to-TGN trafficking. J. Cell Sci..

[26] Rojas R., Van Vlijmen T., Mardones G.A., Prabhu Y., Rojas A.L., Mohammed S., Heck A.J.R., Raposo G., Van Der Sluijs P., Bonifacino J.S. (2008). Regulation of retromer recruitment to endosomes by sequential action of Rab5 and Rab7. J. Cell Biol..

[27] Peña-Llopis S., Vega-Rubin-de-Celis S., Schwartz J.C., Wolff N.C., Tran T.A.T., Zou L., Xie X.-J., Corey D.R., Brugarolas J. (2011). Regulation of TFEB and V-ATPases by mTORC1. EMBO J..

[28] Settembre C., Di Malta C., Polito V.A., Garcia Arencibia M., Vetrini F., Erdin S., Erdin S.U., Huynh T., Medina D., Colella P. (2011). TFEB links autophagy to lysosomal biogenesis. Science.

[29] Matsui T., Jiang P., Nakano S., Sakamaki Y., Yamamoto H., Mizushima N. (2018). Autophagosomal YKT6 is required for fusion with lysosomes independently of syntaxin 17. J. Cell Biol..

[30] Takáts S., Glatz G., Szenci G., Boda A., Horváth G.V., Hegedűs K., Kovács A.L., Juhász G. (2018). Non-canonical role of the SNARE protein Ykt6 in autophagosome-lysosome fusion. PLoS Genet..

[31] Kawamura N., Sun-Wada G.-H., Aoyama M., Harada A., Takasuga S., Sasaki T., Wada Y. (2012). Delivery of endosomes to lysosomes via microautophagy in the visceral endoderm of mouse embryos. Nat. Commun..

[32] Roy S.G., Stevens M.W., So L., Edinger A.L. (2013). Reciprocal effects of *rab7* deletion in activated and neglected T cells. Autophagy.

[33] Mizushima N., Levine B. (2010). Autophagy in mammalian development and differentiation. Nat. Cell Biol..

[34] Pone E.J., Lam T., Lou Z., Wang R., Chen Y., Liu D., Edinger A.L., Xu Z., Casali P. (2015). B cell Rab7 mediates induction of activation-induced cytidine deaminase expression and class-switching in T-dependent and T-independent antibody responses. J. Immunol..

[35] Takahashi K., Mashima H., Miura K., Maeda D., Goto A., Goto T., Sun-Wada G.H., Wada Y., Ohnishi H. (2017). Disruption of small GTPase Rab7 exacerbates the severity of acute pancreatitis in experimental mouse models. Sci. Rep..

[36] Yamaguchi H., Nakagawa I., Yamamoto A., Amano A., Noda T., Yoshimori T. (2009). An initial step of GAS-containing autophagosome-like vacuoles formation requires Rab7. PLoS Pathog..

[37] Yamano K., Fogel A.I., Wang C., van der Bliek A.M., Youle R.J. (2014). Mitochondrial Rab GAPs govern autophagosome biogenesis during mitophagy. Elife.

[38] Yamano K., Wang C., Sarraf S.A., Münch C., Kikuchi R., Noda N.N., Hizukuri Y., Kanemaki M.T., Harper W., Tanaka K. (2018). Endosomal Rab cycles regulate Parkin-mediated mitophagy. eLife.

[39] Jimenez-Orgaz A., Kvainickas A., Nägele H., Denner J., Eimer S., Dengjel J., Steinberg F. (2018). Control of RAB7 activity and localization through the retromer-TBC1D5 complex enables RAB7-dependent mitophagy. EMBO J..

[40] Bucci C., De Luca M. (2012). Molecular basis of Charcot-Marie-Tooth type 2b disease. Biochem. Soc. Trans..

[41] Colecchia D., Stasi M., Leonardi M., Manganelli F., Nolano M., Veneziani B.M., Santoro L., Eskelinen E.L., Chiariello M., Bucci C. (2018). Alterations of autophagy in the peripheral neuropathy Charcot-Marie-Tooth type 2B. Autophagy.

